# The influence of emotional, financial, and control behaviors on physical and sexual violence in intimate relationships during pregnancy: Secondary analysis data

**DOI:** 10.1097/MD.0000000000049750

**Published:** 2026-07-17

**Authors:** Fabiola Vincent Moshi, Keiko Nakamura, Yuri Tashiro, Ayano Miyashita, Runa Katoh, Hideko Sato, Mayumi Ohnishi

**Affiliations:** a Department of Nursing Management and Education, School of Nursing and Public Health, The University of Dodoma, Dodoma, Tanzania; b Department of Global Health Entrepreneurship, The Institute of Science Tokyo, Tokyo, Japan; c Health Data Science Research Unit, Institute for Future Initiatives, The University of Tokyo, Tokyo, Japan; d Graduate School of Biomedical Sciences, Nagasaki University, Nagasaki, Japan.

**Keywords:** control behavior, emotional violence, financial abuse, physical violence, sexual violence

## Abstract

Intimate partner violence (IPV) during pregnancy includes physical, sexual, and emotional abuse, as well as controlling behaviors. However, there is limited information on how emotional, financial, and controlling behaviors affect the occurrence of physical and sexual violence during pregnancy. This study aimed to establish the associations between these types of violence and the occurrence of physical and sexual violence during pregnancy. The study used a secondary analysis of data derived from a larger analytical cross-sectional study. The research was conducted in Central Tanzania between March 4 and April 20, 2024 and included 360 women who had recently delivered. Participants were selected using a 4-stage sampling technique and were interviewed using a semi-structured questionnaire. Participants were selected using a 4-stage sampling technique and interviewed using a semi-structured questionnaire. Bivariate and multivariable logistic regression analyses were conducted using SPSS version 25 to examine factors associated with the outcome variable. The strength of associations was expressed using adjusted odds ratios (AOR) with 95% confidence intervals. Statistical significance was considered at *P* < .05. A total of 36 women (10.0%) experienced physical violence during pregnancy, while 35 women (9.7%) experienced sexual violence. After adjusting for possible confounders, the predictors of physical violence were emotional violence (AOR = 4.316 at 95% CI = 1.488–12.513, *P* = .007) and financial abuse (AOR= 4.356 at 95% CI = 1.711–11.091, *P* = .002), while the predictors of sexual violence were emotional violence (AOR=3.612 at 95% CI = 1.393–9.367, *P* = .008) and controlling behavior (AOR= 4.314 at 95% CI = 1.307–14.241, *P* = .016). The findings demonstrate that multiple forms of intimate partner violence during pregnancy often coexist and collectively compromise maternal and fetal well-being. Comprehensive IPV screening should be integrated into prenatal care, and healthcare providers should be trained to identify all forms of abuse, including emotional, financial, controlling, physical, and sexual violence. Future research should use longitudinal and intervention-based designs to clarify causal pathways, evaluate preventive strategies, and understand how different forms of violence affect maternal and child health outcomes.

## 1. Introduction

Intimate partner violence (IPV) is a substantial public health concern impacting many women, including those who are pregnant. According to the World Health Organization (WHO), approximately 1 in 3 women, totaling around 736 million, experience physical or sexual violence from an intimate partner or sexual violence from a non-partner in their lifetime.^[1]^ In least developed countries, about 37% of women experience physical or sexual IPV, with prevalence reaching up to 50% in some settings.^[2]^ Additionally, women face other types of IPV, including emotional violence, financial/economic abuse, and controlling behaviors.

IPV during pregnancy manifests through physical, sexual, or emotional abuse, along with controlling behaviors.^[3]^ According to the World Health Organization (WHO), emotional violence in an intimate relationship involves actions such as insults, belittling, constant humiliation, intimidation (e.g., destroying personal property), and threats of harm or to take away children.^[4]^ According to WHO, controlling behavior refers to actions intended to limit an individual’s autonomy by isolating them from family and friends, closely monitoring their activities and movements, and restricting their access to financial resources, employment opportunities, education, or healthcare services.^[4]^

Financial abuse refers to behaviors by an intimate partner that control, restrict, or exploit an individual’s access to financial resources, employment, and educational opportunities, thereby compromising their financial security and independence.^[5]^ Beyond ordinary financial disagreements within an intimate relationship, financial abuse involves behaviors in which 1 partner exerts control over household spending, excludes the other partner from financial decision-making, and limits their access to household income and other financial resources.^[6]^

IPV during pregnancy is associated with both fatal and nonfatal adverse health outcomes for the pregnant woman and her baby.^[7]^ This includes direct physical trauma from abuse to the pregnant woman’s body, as well as physiological effects stemming from stress related to current or past abuse, impacting fetal growth and development.^[8]^

The WHO urges countries to demonstrate stronger political will and leadership to comprehensively address violence against women. This involves adopting gender-transformative policies and laws that uphold equality, strengthening health systems to provide survivor-centered care and referrals, and implementing educational programs that challenge discrimination and promote comprehensive sexuality education. It also calls for targeted investment in evidence-based prevention strategies across all levels, alongside improved data collection and high-quality surveys to better measure the diverse forms of violence, particularly among marginalized women.^[1]^

Evidence indicates that, in the context of women’s health and domestic violence, the majority of women experiencing physical abuse during pregnancy had already endured violence before conception.^[9]^ Nonetheless, nearly half of the women surveyed at 3 locations stated that they experienced physical abuse for the first time during pregnancy.^[8]^

Despite the growing body of evidence on the burden of physical and sexual IPV during pregnancy, much less is known about the role of other forms of violence, such as emotional, economic, and controlling behaviors, in shaping women’s vulnerability to physical and sexual abuse. While WHO and other global reports recognize these forms of violence as critical dimensions of IPV, they remain underexplored in the context of pregnancy, particularly in low-resource settings where women already face heightened risks. This limited understanding constrains the development of comprehensive interventions that address the full spectrum of IPV and its cascading effects on maternal and child health. Therefore, this study seeks to fill this gap by examining the influence of emotional, financial, and controlling forms of IPV on the occurrence of physical and sexual violence during pregnancy, to inform more holistic strategies for prevention, early detection, and survivor-centered care.

## 2. Materials and methods

### 2.1. Study design and setting

This study was a secondary analysis of data derived from a larger analytical cross-sectional study conducted among postnatal women in Central Tanzania. The parent study investigated the prevalence and risk factors associated with IPV during pregnancy and has been reported elsewhere.^[10]^ The present analysis was undertaken to address a distinct research objective by examining the influence of emotional violence, financial violence, and controlling behaviors on physical and sexual violence during pregnancy.

The study was conducted in Dodoma and Singida regions, which were selected because they are representative of the demographic, socioeconomic, cultural, and health characteristics of Central Tanzania. Data were collected from 6 health facilities located in urban and rural districts: Makole Health Center, Kondoa District Hospital, and Mpwapwa District Hospital in Dodoma Region, and Sokoine Health Center, Ikungi District Hospital, and Manyoni District Hospital in Singida Region.

### 2.2. Study population

The study included postnatal women attending postnatal clinics between 42 days and 6 months after delivery. Eligible participants were women who had lived with a male partner during pregnancy and provided informed consent. Women who were physically or mentally ill, or whose infants were seriously ill at the time of data collection, were excluded.

### 2.3. Sample size and sampling procedure

The sample size for the parent study was determined using Cochran formula based on an estimated IPV prevalence of 30.3%^[11]^ reported in a previous Tanzanian study. After accounting for a 10% nonresponse rate, a final sample size of 356 participants was obtained.

Participants were recruited from selected district-level health facilities in Dodoma and Singida regions. All eligible postnatal women attending the facilities during the study period were invited to participate. This approach enhanced representativeness and minimized selection bias by ensuring the inclusion of women from diverse demographic and socioeconomic backgrounds.

### 2.4. Data collection

Data were collected using a semi-structured, interviewer-administered questionnaire adapted from previously validated instruments assessing emotional, financial, controlling, physical, and sexual IPV. The questionnaire was reviewed, adapted to the Tanzanian context, translated into Swahili, and pretested before data collection.

Interviews were conducted by 6 trained nurse research assistants fluent in Swahili. To promote privacy and accurate disclosure, interviews were conducted in confidential settings using behaviorally specific questions. Completed questionnaires were checked daily by field supervisors to ensure completeness and consistency.

### 2.5. Variable measurement

Five forms of IPV were assessed: emotional violence, economic violence, controlling behaviors, physical violence, and sexual violence. Measurement items were adapted from standardized instruments and previous studies. Participants were categorized as having experienced a specific form of violence if they reported at least 1 affirmative response within the corresponding domain. Detailed measurement items are provided in the Supplementary Material, Supplemental Digital Content 1.

### 2.6. Data analysis

As this study constituted a secondary analysis of an existing dataset, only variables relevant to the current research objectives were included in the analysis. Data were cleaned and analyzed using IBM SPSS Statistics for Windows, Version 25. Multivariable logistic regression models were fitted to examine the associations between emotional violence, financial violence, controlling behaviors, and the outcomes of physical violence and sexual violence during pregnancy. Covariates included in the adjusted models were selected based on theoretical relevance and findings from bivariate analyses (*P* < .20). Adjusted odds ratios (AORs) and 95% confidence intervals (CIs) were reported, and statistical significance was determined at *P* < .05.

## 3. Results

### 3.1. Socio-demographic characteristic

A total of 360 postnatal mothers participated in the study, yielding a response rate of 100%. The mean age of the women was 27.34 ± 6.34 years (range: 17–45 years), while the mean age of their male partners was 33.68 ± 8.54 years (range: 20–66 years). The average age at marriage was 21.78 ± 4.16 years, ranging from 12 to 40 years. The mean parity was 2.47 ± 1.55 children, with a range of 1 to 9 children.

Most participants were aged 20 to 34 years, had attained primary education, lived in monogamous unions, and were primarily housewives. The majority reported marrying between the ages of 18 and 30 years, initiating antenatal care after the first trimester, and having no history of abortion or preterm birth (Table 1).

**Table 1:** Socio-demographic characteristics of 360 postdelivery mothers in Central Tanzania, 2024.

Variable	Frequency (n)	Percent (%)
Age category woman		
17–19	28	7.8
20–34	276	76.7
35–45	56	15.6
Education level of a woman		
No formal education	15	4.2
Primary education	174	48.3
Secondary education	129	35.8
College or university education	42	11.7
Education level of a partner		
No formal education	11	3.1
Primary education	153	42.5
Secondary education	113	31.4
College or university education	83	23.1
Religion		
Christian	200	55.6
Muslim	160	44.4
Residence		
Rural	163	45.3
Urban	197	54.7
Age at marriage		
12–19 yr	116	32.2
20–40 yr	244	67.8
Type of marriage		
Monogamous	325	90.3
Polygamous	35	9.7
Type of marriage ceremony		
No marriage ceremony	127	35.3
Civil marriage	13	3.6
Religious marriage	172	47.8
Customary marriage	48	13.3
Average monthly income		
<2500 Tshs per day	168	46.7
2500 or more per day	191	53.1
Now living with partner		
Yes	320	88.9
No	40	11.1
Decision maker		
Equally	226	62.8
Male partner	123	34.2
Wife	5	1.4
Others	6	1.7
Occupational status of respondents		
House wife	140	38.9
Farmer	58	16.1
Student	6	1.7
Private employee	55	15.3
Government employee	19	5.3
Occupational of partners		
Farmer	78	21.7
Student	8	2.2
Private employee	104	28.9
Government employee	50	13.9
Others	120	33.3
Alcohol intake in pregnancy		
Yes	32	8.9
No	328	91.1
Male partner’s alcohol habit		
Yes	58	16.1
No	302	83.9
ANC initiation		
1–12 wk	126	35
13–24 wk	194	53.9
25–38 wk	40	11.1
Parity		
Para 1	121	33.6
Para 2–4	196	54.4
More than 4	43	11.9
History of abortion		
Yes	46	12.8
No	314	87.2
History of premature birth		
Yes	16	4.4
No	344	95.6

ANC = antenatal care.

### 3.2. Prevalence of different types of violence during pregnancy

The prevalence of physical violence during pregnancy was 10.0%, with 36 women reporting such experiences, while 324 (90.0%) reported no physical violence. Similarly, 35 women (9.7%) experienced sexual violence during pregnancy, whereas 325 (90.3%) reported no sexual violence. Emotional violence was reported by 154 women (42.8%), while 206 (57.2%) did not experience emotional violence during pregnancy.

**Figure 1.**
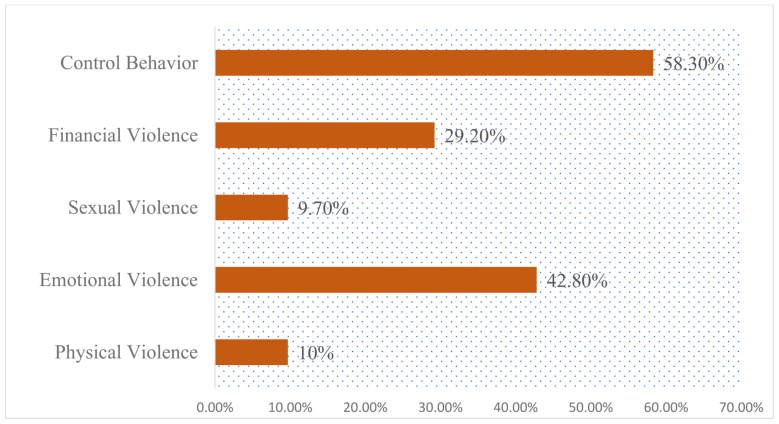
Magnitude of different types of IPV during pregnancy among 360 postdelivery mothers in Central Tanzania. IPV = intimate partner violence.

Financial abuse was experienced by 105 women (29.2%), whereas 255 (70.8%) reported no financial abuse. More than half of the participants, 210 (58.3%), reported being subjected to controlling behaviors by their male partners, while 150 (41.7%) indicated that their partners did not exhibit controlling behaviors during pregnancy (Fig. 1).

**Figure 2.**
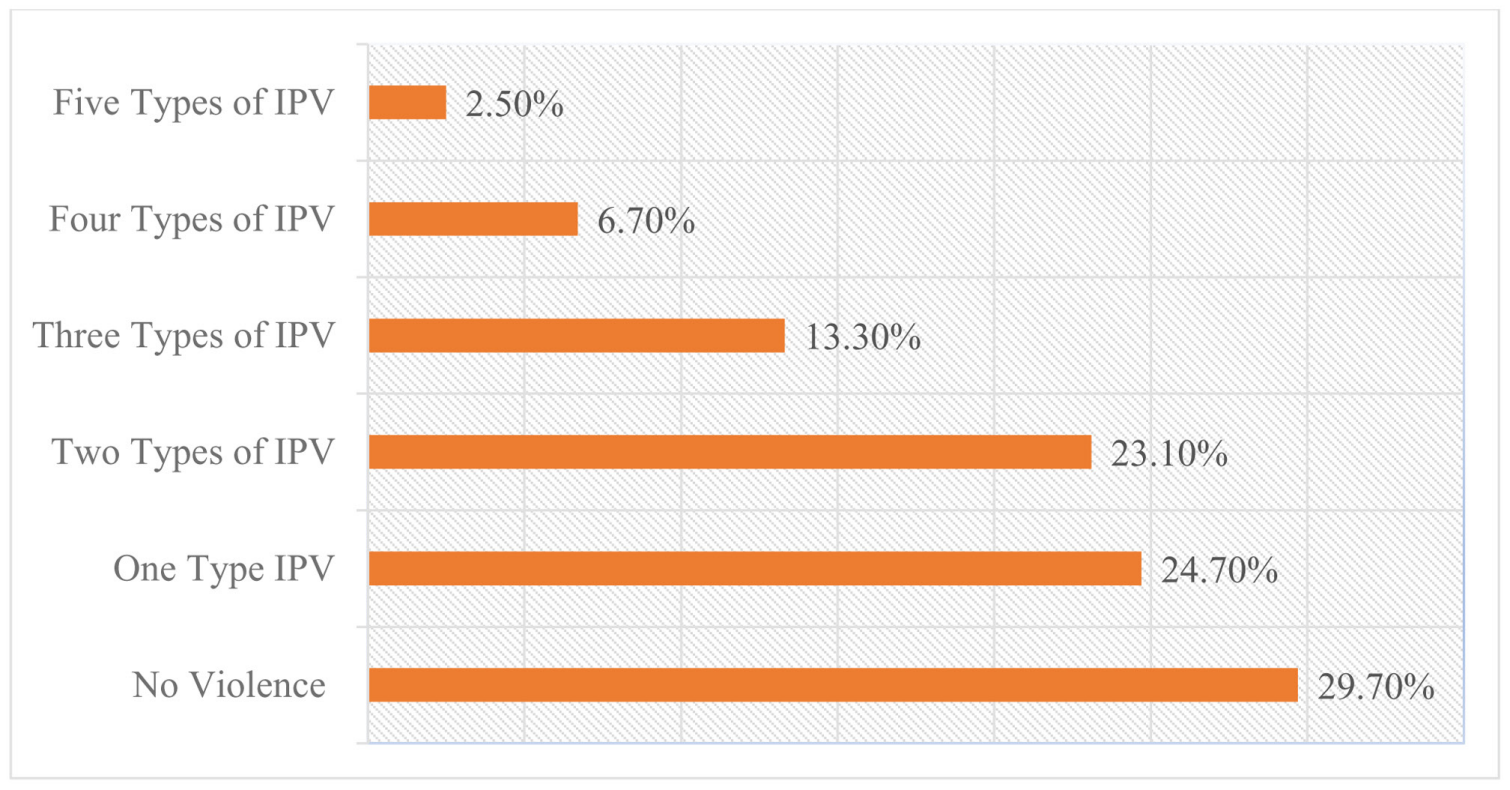
The proportion of women who ever experienced IPV during pregnancy among 360 postdelivery mothers in Central Tanzania. IPV = intimate partner violence.

The study found that a total of 253 (70.3%) of study participants experienced at least 1 type of IPV during pregnancy. Only 107 (29.7%) of interviewed women did not experience any type of IPV during pregnancy. A total of 89 (24.7%) experienced 1 type, 83 (23.1%) experienced 2 types, 48 (13.3%) experienced 3 types, 24 (6.7%) experienced 4 types, and 9 (2.5%) experienced all 5 types of IPV during pregnancy (Fig. 2).

### 3.3. The relationship between women’s characteristics and physical and sexual violence during pregnancy

Variables that had a significant relationship with physical violence include the place of residence of the interviewed women (X^2^ = 12.881, *P* = .025), the occupation of their male partners (X^2^ = 12.158, *P* = .016), household decision-making (X^2^ = 8.720, *P* = .033), emotional violence (X^2^ = 30.685, *P* < .001), financial abuse (X^2^ = 35.892, *P* < .001), and controlling behavior (X^2^ = 10.286, *P* = .001). Variables with a significant relationship with sexual violence were place of residence (X^2^ = 15.905, *P* = .007), emotional violence (X^2^ = 21.944, *P* < .001), financial abuse (X^2^ = 17.840, *P* < .001), and controlling behavior (X^2^ = 11.959, *P* = .001; Table 2).

**Table 2:** The relationship between women’s characteristics and Physical, and Sexual Violence during pregnancy among 360 postdelivery mothers in Central Tanzania, 2024.

Variables	Physical violence		X^2^	*P*-value	Sexual violence		X^2^	*P*-value
	No n(%)	Yes n(%)			No n(%)	Yes n(%)		
ANC initiation			0.524	.769			3.011	.222
1–12 wk	115 (91.3)	11 (8.7)			110 (87.3)	16 (12.7)		
13–24 wk	174 (89.7)	20 (10.3)			180 (92.8)	14 (7.2)		
25–38 wk	35 (87.5)	5 (12.5)			35 (87.5)	5 (12.5)		
Place of residence			12.811	.025			15.905	.007
Dodoma City	50 (79.4)	13 (20.6)			53 (84.1)	10 (15.9)		
Kondoa District	78 (92.9)	6 (7.1)			77 (91.7)	7 (8.3)		
Mpwapwa District	80 (93)	6 (7)			82 (95.3)	4 (4.7)		
Manyoni District	40 (90.9)	4 (9.1)			40 (90.9)	4 (9.1)		
Ikungi District	27 (84.4)	5 (15.6)			24 (75)	8 (25)		
Singida Municipal	49 (96.1)	2 (3.9)			49 (96.1)	2 (3.9)		
Religion			3.125	.077			0.310	.578
Christian	175 (87.5)	25 (12.5)			179 (89.5)	21 (10.5)		
Muslim	149 (93.1)	11 (6.9)			146 (91.3)	14 (8.8)		
Residence			1.705	.192			2.203	.138
Rural	143 (87.7)	20 (12.3)			143 (87.7)	20 (12.3)		
Urban	181 (91.9)	16 (8.1)			182 (92.4)	15 (7.6)		
Dowry/bride price payment			.639	.424			0.230	.632
Yes	253 (90.7)	26 (9.3)			253 (90.7)	26 (9.3)		
No	71 (87.7)	10 (12.3)			72 (88.9)	9 (11.1)		
Type of marriage ceremony			1.518	.678			1.545	.672
No ceremony	114 (89.8)	13 (10.2)			115 (90.6)	12 (9.4)		
Civil marriage	12 (92.3)	1 (7.7)			13 (100)	0 (0.0)		
Religious marriage	157 (91.3)	15 (8.7)			154 (89.5)	18 (10.5)		
Customary marriage	41 (85.4)	7 (14.6)			43 (89.6)	5 (10.4)		
Now living with partner			1.250	.264			0.253	.615
Yes	290 (90.6)	30 (9.4)			288 (90)	32 (10)		
No	34 (85)	6 (15)			37 (92.5)	3 (7.5)		
Occupation of partners			12.158	.016			6.804	.147
Farmer	70 (89.7)	8 (10.3)			68 (87.2)	10 (12.8)		
Student	7 (87.5)	1 (12.5)			7 (87.5)	1 (12.5)		
Private employee	86 (82.7)	18 (17.3)			90 (86.5)	14 (13.5)		
Government employee	45 (90)	5 (10)			45 (90)	5 (10)		
Others	116 (96.7)	4 (3.3)			115 (95.8)	5 (4.2)		
Alcohol intake in pregnancy			0.244	.621			0.005	.945
Yes	28 (87.5)	4 (12.5)			29 (90.6)	3 (9.4)		
No	296 (90.2)	32 (9.8)			296 (90.2)	32 (9.8)		
Husband alcohol habit			1.105	.293			0.434	.51
Yes	50 (86.2)	8 (13.8)			51 (87.9)	7 (12.1)		
No	274 (90.7)	28 (9.3)			274 (90.7)	28 (9.3)		
History of abortion			3.201	.074			1.814	.178
Yes	38 (82.6)	8 (17.4)			39 (84.8)	7 (15.2)		
No	286 (91.1)	28 (8.9)			286 (91.1)	28 (8.9)		
Parity categorized			2.417	.299			2.407	.3
Para 1	112 (92)	9 (7.4)			110 (90.9)	11 (9.1)		
Para 2–4	172 (87.8)	24 (12.2)			179 (91.3)	17 (8.7)		
More than 4	40 (93)	3 (7)			36 (83.7)	7 (16.3)		
Decision maker			8.720	.033			1.830	.608
Equally	210 (92.9)	16 (7.1)			206 (91.2)	20 (8.8)		
Husband	103 (83.7)	20 (16.3)			109 (88.6)	14 (11.4)		
Wife	5 (100)	0 (0.0)			4 (80)	1 (20)		
Others	6 (100)	0 (0.0)			6 (100)	0 (0.0)		
Emotional violence			30.685	<.001			21.944	<.001
No	201 (97.6)	5 (2.4)			199 (96.6)	7 (3.4)		
Yes	123 (79.9)	31 (20.1)			126 (81.8)	28 (18.2)		
Financial abuse			35.892	<.001			17.840	<.001
No	245 (96.1)	10 (3.9)			241 (94.5)	14 (5.5)		
Yes	79 (75.2)	26 (24.8)			84 (80)	21 (20)		
Control behavior			10.286	.001			11.959	.001
No	144 (96)	6 (4)			145 (96.7)	5 (3.3)		
Yes	180 (85.7)	30 (14.3)			180 (85.7)	30 (14.3)		

ANC = antenatal care.

### 3.4. The influence of emotional, financial, and control behaviors on physical violence during pregnancy

Predictors of physical violence were emotional violence AOR = 4.316 at 95% CI = 1.488–12.513, *P* = .007 and financial abuse AOR = 4.356 at 95% CI = 1.711–11.091, *P* = .002 (Table 3).

**Table 3:** The influence of emotional, financial, and control behaviors on physical violence during pregnancy among 360 postdelivery mothers in Central Tanzania, 2024.

Variables	OR (95% CI)	*P*-value	AOR (95% CI)	*P*-value
Place of residence				
Dodoma City	1		1	
Kondoa District	0.296 (0.106–0.829)	.021	1.487 (0.388–5.699)	.562
Mpwapwa District	0.288 (0.103–0.808)	.018	0.888 (0.231–3.404)	.862
Manyoni District	0.385 (0.116–1.271)	.117	1.784 (0.374–8.504)	.467
Ikungi District	0.712 (0.229–2.211)	.557	2.947 (0.659–13.177)	.157
Singida Municipal	0.157 (0.034–0.732)	.018	0.435 (0.082–2.304)	.328
Religion				
Christian	1.935 (0.921–4.064)	.081	2.2538 (0.825–6.151)	.113
Muslim	1		1	
Occupation of partners				
Farmer			1	
Student	1.25 (0.136–11.501)	.844	0.743 (0.053–10.437)	.825
Private employee	1.831 (0.752–4.462)	.183	2.056 (0.647–6.541)	.222
Government employee	0.972 (0.299–3.159)	.963	0.806 (0.200–3.255)	.763
Others	0.302 (0.088–1.039)	.057	0.487 (0.121–1.950)	.309
History of abortion				
Yes	2.15 (0.914–5.059)	.079	2.002 (0.730–5.489)	.177
No	1		1	
Emotional violence				
No	1		1	
Yes	10.132 (3.837–26.750)	<.001	4.316 (1.488–12.513)	.007
Financial abuse				
No	1		1	
Yes	8.063 (3.725–17.452)	<.001	4.356 (1.711–11.091)	.002
Control behavior				
No	1		1	
Yes	4 (1.621–9.873)	.003	1.923 (0.630–5.870)	.251

AOR = adjusted odds ratio, CI = confidence interval, OR = odds ratio.

### 3.5. The influence of emotional, financial, and control behaviors on sexual violence during pregnancy

Predictors of sexual violence were emotional violence (AOR = 3.612 at 95% CI = 1.393–9.367, *P* = .008) and control behavior AOR = 4.314 at 95% CI = 1.307–14.241, *P* = .016 (Table 4).

**Table 4:** The influence of emotional, financial, and control behaviors on sexual violence during pregnancy among 360 postdelivery mothers in Central Tanzania, 2024.

Variables	OR (95% CI)	*P*-value	AOR (95%CI)	*P*-value
Place of residence				
Dodoma City	1		1	
Kondoa District	0.482 (0.172–1.346)	.164	0.842 (0.277)	.762
Mpwapwa District	0.259 (0.077–0.867)	.028	0.4 (0.105–1.528)	.18
Manyoni District	0.53 (0.155–1.813)	.312	1.228 (0.318–4.741)	.766
Ikungi District	1.767 (0.620–5.035)	.287	5.902 (1.560–22.322)	.009
Singida Municipal	0.216 (0.045–1.037)	.056	0.474 (0.092–2.459)	.374
Emotional violence				
No	1		1	
Yes	6.317 (2.679–14.896)	<.001	3.612 (1.393–9.367)	.008
Financial abuse				
No	1		1	
Yes	4.304 (2.094–8.845)	<.001	1.905 (0.785–4.627)	.154
Control behavior				
No	1		1	
Yes	4.833 (1.829–12.772)	.001	4.314 (1.307–14.241)	.016

AOR = adjusted odds ratio, CI = confidence interval, OR = odds ratio.

## 4. Discussion

The study revealed a high prevalence of IPV during pregnancy, with 70.3% of women experiencing at least 1 form of IPV. This prevalence is higher than reports from other settings, including Ethiopia (37.5%) and Belgium (10.6%).^[12,13]^ The high burden observed may be attributed to the inclusion of multiple forms of IPV, particularly controlling behaviors and financial abuse, highlighting the widespread nature of violence against pregnant women and the need for comprehensive screening and intervention strategies.

The study further found that 10% of pregnant women experienced physical violence and 9.7% experienced sexual violence. The prevalence of physical violence is comparable to global estimates (9.2%) and findings from Belgium (10.6%),^[13,14]^ while the prevalence of sexual violence is higher than the global estimate of 5.5%.^[14]^ These findings indicate that physical and sexual violence remain significant public health concerns during pregnancy. The relatively high prevalence of sexual violence may reflect coercive control, unequal power relations, and sociocultural norms that reinforce male dominance and limit women’s autonomy.^[15,16]^

Furthermore, nonphysical forms of intimate partner violence were prevalent during pregnancy, with 42.8% of women reporting emotional violence, 29.2% experiencing financial abuse, and 58.3% being subjected to controlling behaviors. These findings are consistent with previous studies identifying emotional abuse and controlling behaviors as common manifestations of coercive control that restrict women’s autonomy and decision-making power.^[15,17,18]^ The increased emotional abuse could be attributed by the fact that pregnancy can bring about increased stress and anxiety for both partners.^[3]^ Some individuals may lack healthy coping mechanisms and resort to emotional abuse as a way to manage their own stress and feelings of inadequacy. Another reason could be that some abusers may avoid physical violence during pregnancy due to the visible consequences or potential harm to the unborn child. Instead, they may turn to emotional abuse, which can be equally damaging but less immediately apparent.^[19]^ Additionally, pregnant women may experience increased emotional sensitivity and vulnerability, making the impact of emotional violence more profound.^[20]^ Male partners may exploit this increased sensitivity to inflict greater psychological harm.

After adjusting for potential confounders, emotional violence and financial abuse remained significant predictors of physical violence during pregnancy. Women who experienced emotional violence were more than 4 times more likely to experience physical violence, while those subjected to financial abuse had a similarly increased risk. These findings support previous evidence suggesting that different forms of intimate partner violence often coexist and are manifestations of broader patterns of coercive control within relationships.^[15,16]^

Emotional abuse may diminish women’s self-esteem and ability to seek help, while financial abuse increases economic dependency and vulnerability, creating conditions that facilitate physical violence. Similar evidence suggests that the coexistence of emotional and physical violence increases the risk of adverse mental health outcomes, including anxiety and depression.^[21]^ Furthermore, financial abuse is frequently used to maintain power and control over women and may escalate into physical violence when economic control alone is insufficient to achieve dominance.^[22]^ The findings emphasize the importance of recognizing emotional and economic abuse as early warning signs of escalating violence during pregnancy.

Similarly, emotional violence and controlling behaviors were significant predictors of sexual violence during pregnancy. Women who experienced emotional violence were more than 3 times as likely to report sexual violence, while those exposed to controlling behaviors were over 4 times as likely to experience sexual abuse. These findings suggest that sexual violence rarely occurs in isolation and is often embedded within broader patterns of emotional abuse and coercive control. Emotional abuse may increase women’s vulnerability by undermining their self-esteem, confidence, and ability to resist unwanted sexual advances, thereby increasing the risk of sexual victimization during pregnancy.

Likewise, controlling behaviors may increase the likelihood of sexual violence by restricting women’s autonomy and limiting their ability to negotiate sexual relationships. Such behaviors are often used by perpetrators to assert power and dominance, extending control over various aspects of a woman’s life, including her sexual and reproductive choices. These findings are consistent with previous studies that identify coercive control as a central feature of intimate partner violence and a key driver of sexual abuse.^[15,16]^ Similar observations by Dichter et al^[17]^ further demonstrate that controlling behaviors frequently coexist with sexual violence as part of a broader pattern of abuse. Together, these findings underscore the importance of screening for emotional abuse and controlling behaviors during antenatal care as potential indicators of sexual violence.

While this study is comprehensive, its cross-sectional design prevents establishing causal relationships among the reported factors, and temporality between predictor and outcome variables cannot be assured. To mitigate this limitation, we conducted rigorous regression analyses, controlling for potential confounders, which enhances the reliability of the findings despite the inherent constraints. Given the sensitivity of the topic and cultural beliefs surrounding IPV, there remains a possibility of underreporting. Therefore, we recommend future qualitative studies to explore this issue in greater depth and uncover nuanced perspectives.

## 5. Conclusion

These findings underscore the complex interplay of various types of IPV during pregnancy, impacting maternal and fetal well-being. Addressing these factors is crucial for mitigating the adverse health outcomes associated with IPV in this vulnerable population. It is recommended to implement comprehensive screening protocols for IPV during prenatal care visits. Healthcare providers should receive training to recognize signs of emotional violence, financial abuse, and controlling behaviors, alongside physical and sexual violence. From a scholarly perspective, future research should employ longitudinal and intervention-based study designs to establish causal pathways, evaluate the effectiveness of preventive strategies, and deepen understanding of how different forms of violence collectively influence maternal and child health outcomes.

## Acknowledgments

We gratefully acknowledge the Matsumae International Foundation for sponsoring the fellowship, the Institute of Science Tokyo (formerly Tokyo Medical and Dental University) for hosting the research fellow, and the University of Dodoma for granting study leave. We also thank the research assistants for their support in data collection and, most importantly, the participants for their invaluable contributions to this study.

## Author contributions

**Conceptualization:** Fabiola Vincent Moshi, Keiko Nakamura.

**Data curation:** Fabiola Vincent Moshi, Keiko Nakamura.

**Formal analysis:** Fabiola Vincent Moshi, Keiko Nakamura, Hideko Sato.

**Methodology:** Fabiola Vincent Moshi, Keiko Nakamura.

**Supervision:** Keiko Nakamura, Yuri Tashiro, Ayano Miyashita, Hideko Sato, Mayumi Ohnishi.

**Validation:** Keiko Nakamura.

**Writing – original draft:** Fabiola Vincent Moshi.

**Writing – review & editing:** Fabiola Vincent Moshi, Keiko Nakamura, Yuri Tashiro, Ayano Miyashita, Runa Katoh, Hideko Sato, Mayumi Ohnishi.


