# Supplementary material for: The influence of emotional, financial, and control behaviors on physical and sexual violence in intimate relationships during pregnancy: Secondary analysis data
Source: Medicine (Baltimore). 2026 Jul 17;105(29):e49750. doi: 10.1097/MD.0000000000049750 (PMC13384700; doi:10.1097/MD.0000000000049750)
Supplement: Supplementary file 1 [file medi-105-e49750-s001.docx]

**Questions used to assess the five forms of IPV**

1. **Physical violence**

To assess experiences of physical violence during pregnancy, six specific questions were utilized:

1. Has your partner ever slapped you or thrown something at you that could hurt you during pregnancy?
2. Has your partner ever pushed, shoved, or pulled your hair during pregnancy?
3. Has your partner ever hit you with his fist or with something else that could hurt you during pregnancy?
4. Has your partner ever beaten you in the abdomen during pregnancy?
5. Has your partner ever choked or burnt you on purpose during pregnancy?
6. Has your partner ever threatened to use or actually used a gun, knife, or any other weapon against you during pregnancy?

The response was dichotomized into either Yes or No (Deshpande & Lewis-O’connor, 2013)

1. **Emotional Violence**

To assess pregnant women’s experiences of emotional violence, four targeted questions were used:

1. Has your partner ever insulted/made feel bad about self during pregnancy?
2. Has your partner ever belittled or humiliated in front of other people during pregnancy?
3. Has your partner ever scared or intimidated on purpose during pregnancy?
4. Has your partner ever threatened you when visiting friends/family during pregnancy?

The response to these questions were dichotomized to Yes or No

1. **Sexual Violence**

To assess pregnant women’s experiences of sexual violence, three targeted questions were employed:

1. Has your partner ever physically forced you to have sexual intercourse during pregnancy?
2. Has your partner ever having unwanted sexual intercourse because of fear from the partner during pregnancy?
3. Has your partner ever forced you to do something sexual that is degrading or humiliating during pregnancy?

The response to these questions were dichotomized to Yes or No

1. **Financial or economic abuse**

The assessment of financial abuse was conducted using nine specific questions, which included:

1. Have you ever been denied access to money or financial resources by your partner during pregnancy?
2. Has your partner ever controlled all the finances in the relationship without your input or knowledge during pregnancy?
3. Do you feel pressured or forced to account for every cent spent, even on basic necessities during pregnancy?
4. Have you been prevented from working or pursuing education to maintain financial independence during pregnancy?
5. Has your partner coerced you into signing financial documents or contracts against your will during pregnancy?
6. Do you fear repercussions or retaliation if you spend money without your partner's permission during pregnancy?
7. Have you experienced threats of financial abandonment or homelessness if you don't comply with your partner's demands during pregnancy?
8. Have you ever been manipulated or guilt-tripped into giving your partner money or taking on their financial responsibilities during pregnancy?
9. Do you feel trapped in the relationship due to financial dependence or lack of resources to leave during pregnancy?

The response to these questions were dichotomized to Yes or No

1. **Controlling Behaviors**

Seven questions were employed to assess partners' controlling behaviors, including:

1. Has your partner ever tried to keep you from seeing friends during pregnancy?
2. Has your partner ever tried to restrict contacts with your family members during pregnancy?
3. Has your partner ever insisted to know where you are all the time during pregnancy?
4. Has your partner ever ignored you and treated you indifferently during pregnancy?
5. Has your partner ever got angry if you speak with other man during pregnancy?
6. Has your partner ever become suspicious that you were unfaithful during pregnancy?
7. Has your partner ever expect you to ask permission when you wanted to seek health care during pregnancy?
